# Editorial: Obstructive sleep apnea syndrome (OSAS). What's new?

**DOI:** 10.3389/fmed.2022.1009410

**Published:** 2022-09-15

**Authors:** Barbara Ruaro, Elisa Baratella, Marco Confalonieri, Caterina Antonaglia, Francesco Salton

**Affiliations:** ^1^Pulmonology Unit, Department of Medical Surgical and Health Sciences, University Hospital of Cattinara, University of Trieste, Trieste, Italy; ^2^Department of Radiology, Department of Medicine, Surgery and Health Science, University of Trieste, Trieste, Italy

**Keywords:** OSAS, CPAP, polysomnography, sleep disorders, apnea syndrome

This Research Topic entitled “*Obstructive sleep apnea syndrome (OSAS). What's new?”*, involving authors from different specializations and numerous countries, confirms that OSAS is a hot topic. OSA syndrome is an airway obstruction (i.e. complete or partial) with numerous etiologies (1–4). Different papers have demonstrated that the prevalence of OSAS is 2–4% in men and 1–2% in women of average age. The reference tools for OSAS diagnosis are clinical polysomnography or nocturnal portable multi-channel monitoring. Frequently, continuous positive airway pressure (CPAP) therapy is the first treatment for a patient (5, 6). Long-term CPAP treatment may present limited compliance, and there is no unanimous opinion on other alternative treatments for OSAS in literature on the subject. This special issue discusses several of these “unmet needs”.

In the first article (Braghiroli et al.) of the collection, the authors compared 118 OSAS patients with different disease severity, using a standard monitoring system (Nox T3) and a low-cost device (AirgoTM) with an elastic band and a small recorder, which is lightweight and comfortable for the patient. The data showed that AirgoTM has an excellent level of sensitivity, specificity, positive and negative predicted value, and accuracy. The authors reported that AirgoTM is a reliable tool for screening suspected OSAS patients, the device was well tolerated by the subjects, and that it is based on an automatic analysis that can help physicians to increase the selection of possible candidates for treatment.

The second article (Conte et al.) evaluates a simplified non-invasive screening system for improving the diagnosis of OSAS. The authors used the original Berlin Questionnaire, simplified to a set of questions specifically included by the authors' group that complemented the socio-demographic, clinical, and medical history framework of the participants, to investigate the impact of OSAS on health care systems. In conclusion, the authors demonstrated that their reduced version of the Berlin questionnaire seems capable of reproducing the outcome of the original questionnaire. Furthermore, the decrease in the number of questions may be useful for screening possible cases of OSAS in situations where the time it takes to complete the questionnaire needs to be as short as possible.

In another article (Cai et al.) the researchers selected 74 OSAS patients and classified them into mild, moderate, and severe diseases. In addition, the authors selected 20 subjects as a control group. The investigators evaluated the serum levels of liver fibrosis markers using electrochemiluminescence immunoassay. The researchers exposed hepatic stellate cells to intermittent hypoxia (IH) or normoxia (RA). The authors observed that serum liver fibrosis markers were positively correlated with the apnea-hypopnea index (AHI) but negatively correlated with lowest saturation oxygen (LSaO2) respectively. Lastly, the experiments support the view that OSAS might either directly or indirectly trigger or exacerbate liver fibrosis by IH-related pathways.

The aim of the fourth study (Stavrou, Koutedakis et al.) was to investigate the effect of different pillows [own pillow (OP), foam-memory pillow (MFP), generic laboratory pillow (LP)] on selected polysomnography (PSG) evaluations in 32 OSAS patients. Interestingly, the study indicates that pillow type and use, which is often uncontrolled in OSAS assessments, correlates with several PSG parameters and a snoring subtype of the syndrome.

In the fifth paper (Chung et al.) the authors demonstrated in 62 OSAS patients with overnight oxygen desaturation (sPO2 < 90%) that neck CT with computational fluid dynamics evaluation of airway pressure and airflow velocity may offer a quick prediction of moderate to severe OSAS.

The sixth article undertakes an evaluation of possible correlations between brain-derived neurotrophic factor (BDNF), OSAS, and endothelial dysfunction (Makhout et al.). This study included 103 children, of which 20 had OSAS, all with obesity, aged 8 to 18 years. The authors also investigated the possible effect of weight loss on serum BDNF levels, as BDNF has a significant part in the regulation of food intake and body weight. In conclusion, the authors demonstrated that BDNF levels were similar in children with obesity, with and without OSA, indicating that BDNF concentration is not influenced by OSAS. The authors observed an effect of OSAS and endothelial function on BDNF levels.

The seventh article (Gao et al.) is a review that explores the evidence of duration and quality of sleep as evaluated by multiple health outcomes. The authors included 85 meta-analyses and 36 health outcomes. The researchers underlined the association between short sleep and an improvement in the risk of becoming overweight and/or obese; and poor sleep quality and an increased risk of both mellitus and gestational diabetes. They reported that the correlation of long sleep with increased risk of all-cause mortality (stroke, dyslipidaemia, mortality of coronary heart disease, stroke mortality, and the developing or dying of stroke) were graded as highly suggestive.

The eighth study (Hu et al.) evaluated the prognostic factors and survival rates of 90 lung cancer patients with OSAS by nomogram. There were significant differences in sex, apnea hypopnea index (AHI), Tumor Node Metastasis (TNM), coronary heart disease, lowest arterial oxygen saturation, and oxygen desaturation index (ODI4) between the lung cancer subgroup and lung cancer with the OSAS subgroup. In the lung cancer group with OSAS, six factors (i.e. age, AHI, TNM, cancer types, BMI, and ODI4) were independent prognostic factors, and a nomogram model was established based on these parameters. The authors demonstrated that the nomogram could predict the prognosis of patients and guide personalized treatment regimes.

The ninth paper (Stavrou, Astara et al.) reviews literature on the altered systematic pathophysiologic mechanisms in OSAS subjects and proposes a correct exercise program for all subjects. The interesting results of this study indicate that exercise prevents a dysregulation of both daytime and nighttime cardiovascular autonomic function, reduces body weight, and halts the onset and progress of insulin resistance, while it ameliorates excessive daytime sleepiness, cognitive decline, and mood disturbances, resulting in an improvement in the quality of sleep and life.

The tenth article (Duan et al.) examined the correlations of OSAS risk with depression, anxiety, and life events in 10,287 Chinese subjects. The Berlin Questionnaire (BQ) was used to ascertain the level of OSAS, while Depression Scale (CES-D) and Zung's self-rating anxiety scale (SAS) were used to define depression and anxiety. The results indicated that depression and anxiety, especially when both co-occur at high levels, were correlated with an increased risk of OSAS. However, adverse life events were not correlated with any risk of OSAS. The authors advised increasing attention on depression and anxiety in OSAS patients.

In the last article, the authors (Chen et al.) illustrate a strategy for realizing a systematic review, exploring the association between sleep-disordered breathing and periodontal diseases, a topic of controversy and clinical significance. The authors underline that the results can provide evidence for the development of relevant prevention and treatment strategies.

Overall, this collection of papers underlines the importance of assessing the risk of obstructive sleep apnea syndrome, as this syndrome is linked with a high risk of hypertension, cardiovascular diseases, daytime sleepiness, home and work-related accidents, and a consequent worsening of life quality. Numerous studies stress the importance of praecox diagnosis and a multidisciplinary approach in addressing all these situations.

## Author contributions

BR, EB, and FS compiled the manuscript. MC and CA undertook final amendments and approved the final version. All authors contributed to the article and approved the submitted version.

## Conflict of interest

The authors declare that the research was conducted in the absence of any commercial or financial relationships that could be construed as a potential conflict of interest.

## Publisher's note

All claims expressed in this article are solely those of the authors and do not necessarily represent those of their affiliated organizations, or those of the publisher, the editors and the reviewers. Any product that may be evaluated in this article, or claim that may be made by its manufacturer, is not guaranteed or endorsed by the publisher.

## References

[1] Levrini L Sacchi F Milano F Polimeni A Cozza P Bernkopf E. Italian recommendations on dental support in the treatment of adult obstructive sleep apnea syndrome (OSAS). Ann Stomatol (Roma). (2016) 6:81–6. 10.11138/ads/2015.6.3.081 26941893 PMC4755685

[2] Ma B Li Y Wang X Du L Wang S Ma H. Association between abdominal adipose tissue distribution and obstructive sleep apnea in Chinese obese patients. Front Endocrinol (Lausanne). (2022) 13:847324. 10.3389/fendo.2022.847324 35399929 PMC8988152

[3] Ruaro B Salton F Braga L Wade B Confalonieri P Volpe MC. The history and mystery of alveolar epithelial type ii cells: focus on their physiologic and pathologic role in lung. Int J Mol Sci. (2021) 22:2566. 10.3390/ijms22052566 33806395 PMC7961977

[4] Antonaglia C Passuti G Giudici F Salton F Ruaro B Radovanovic D. Low arousal threshold: a common pathophysiological trait in patients with obstructive sleep apnea syndrome and asthma. Sleep Breath. (2022). 10.1007/s11325-022-02665-4. [Epub ahead of print]. 35907116 PMC10226905

[5] Portel L Parrat E Nocent-Ejnaini C Mangiapan G Prud'homme A Oster JP. Phenotyping to target obstructive sleep apnoea syndrom (OSAS) in adults patients with severe asthma. Respir Med Res. (2022) 82:100888. 10.1016/j.resmer.2022.100888 35691215

[6] Tingting X Danming Y Xin C. Non-surgical treatment of obstructive sleep apnea syndrome. Eur Arch Otorhinolaryngol. (2018) 275:335–46. 10.1007/s00405-017-4818-y 29177626

